# Nonfatal Assaults and Homicides Among Adults Aged ≥60 Years — United States, 2002–2016

**DOI:** 10.15585/mmwr.mm6813a1

**Published:** 2019-04-05

**Authors:** J. E. Logan, Tadesse Haileyesus, Allison Ertl, Whitney L. Rostad, Jeffrey H. Herbst

**Affiliations:** ^1^Division of Violence Prevention, National Center for Injury Prevention and Control, CDC; ^2^Division of Analysis, Research, and Practice Integration, National Center for Injury Prevention and Control, CDC.

Since interpersonal violence was recognized as a public health problem in the 1970s, much attention has focused on preventing violence among young persons and intimate partners (*1*). Violence directed against older adults (≥60 years) has received less attention, despite the faster growth of this population than that of younger groups (*2*). Using data from the National Electronic Injury Surveillance System–All Injury Program (NEISS-AIP) and the National Vital Statistics System (NVSS), CDC analyzed rates of nonfatal assaults and homicides against older adults during 2002–2016. Across the 15-year period, the nonfatal assault rate increased 75.4% (from 77.7 to 136.3 per 100,000) among men, and from 2007 to 2016, increased 35.4% (from 43.8 to 59.3) among women. From 2010 to 2016, the homicide rate increased among men by 7.1%, and a 19.3% increase was observed from 2013 to 2016 among men aged 60–69 years. Growth in both the older adult population and the rates of violence against this group, especially among men, suggests an important need for violence prevention strategies (*3*). Focusing prevention efforts for this population will require improved understanding of magnitude and trends in violence against older adults.

In 2017, older adults accounted for 22% of the U.S. population, surpassing children and adolescents aged 0–14 years (19%); this percentage is expected to reach 28% by 2050 (*2*). Many older adults require care and are vulnerable to violence perpetrated by a caregiver or someone they trust (*4*).

To assess nonfatal assault trends, data from the NEISS-AIP system, operated by the U.S. Consumer Product Safety Commission in collaboration with CDC, were analyzed. NEISS-AIP collects data on approximately 500,000 nonfatal injury–related visits annually from a nationally representative sample of hospital emergency departments (EDs), and data are weighted by the inverse probability of selection to produce national estimates (*5*). Trained coders classify injuries into categories by intent (i.e., unintentional, assault, self-inflicted, and legal intervention) (*6*). Nonfatal assault injuries were limited to those resulting from physical violence by one or more persons; sexual assaults and injuries from legal intervention were excluded. Rates were based on weighted data from 11,373 nonfatal assault–related injuries among older adults treated during 2002 to 2016.

NVSS data were used to analyze homicide trends. NVSS receives information on manner and cause of death and decedent demographics from death certificates provided by vital statistics registration systems across the United States. Homicides were limited to injury deaths with *International Classification of Diseases, Tenth Revision* underlying cause of death codes X85–Y09, Y87.1, and U01–U02.*

Trends were analyzed using SAS (version 9.4; SAS Institute) and were stratified by 10-year age groups (60–69, 70–79, and ≥80 years). Crude rates per 100,000 population per year were calculated using U.S. Census bridged-race resident population estimates.^†^ The estimated overall average annual nonfatal assault rate and the observed average annual homicide rate, as well as rates by sex and age group, were computed. Joinpoint^§^ (version 4.6.0; National Cancer Institute) regression was used to model rates^¶^ and test trend significance. Annual percentage change estimates indicate magnitude and direction of trends across the study period. Mechanisms of nonfatal injury and homicide reported by NEISS-AIP and NVSS are also described.

From 2002 to 2016, an estimated 643,191 nonfatal assault victims were treated in EDs, and 19,059 homicides occurred (Table). Compared with women, men experienced higher rates of both nonfatal assaults (107.8 versus 50.4 per 100,000) and homicides (3.16 versus 1.53). On average, the highest rates of nonfatal assaults (147.4) and homicides (3.58) were perpetrated against men aged 60–69 years. The nonfatal assault rate was lowest among women aged 70–79 years (36.0), and the homicide rate was lowest among women aged 60–69 years (1.40).

**TABLE:** Number and rate of estimated* nonfatal assaults and actual fatal assaults (homicides) among persons aged ≥60 years, by sex and age group — United States, 2002–2016

Sex/Age group (yrs)	No. of sample cases	No. of injuries^†,§^ (%)	Average annual rate^†^ (95% CI)	No. of joinpoints	Joinpoint year range	APC	Modeled rate^¶^ range	Overall % change in modeled rate range^¶^
**Nonfatal assault**								
Total, ≥60	11,373	643,191 (100.0)	75.9 (60.6–91.2)	1	2002–2008	0.26	63.8–64.8	1.6
					2008–2016	5.47**	64.8–99.2	53.1
Men, ≥60	7,452	405,527 (63.0)	107.8 (81.3–134.4)	0	2002–2016	4.10**	77.7–136.3	75.4
Women, ≥60	3,921	237,664 (37.0)	50.4 (43.2–57.7)	1	2002–2007	−2.74	50.4–43.8	−13.1
					2007–2016	3.41**	43.8–59.3	35.4
Men, 60–69	5,629	296,789 (73.2)	147.4 (107.9–186.9)	0	2002–2016	4.66**	100.5–190.1	89.2
Men, 70–79	1,353	80,939 (20.0)	70.6 (54.4–86.7)	0	2002–2016	2.06**	60.5–80.5	33.1
Men, ≥80	470	27,799 (6.9)	46.3 (38.0–54.7)	0	2002–2016	1.12	42.0–49.0	16.7
Women, 60–69	2,478	147,685 (62.1)	66.3 (56.1–76.6)	0	2002–2016	2.29**	55.0–75.6	37.5
Women, 70–79	858	51,430 (21.6)	36.0 (30.1–41.9)	0	2002–2016	−0.53	38.0–35.3	−7.1
Women, ≥80	585	38,550 (16.2)	36.5 (31.0–42.0)	0	2002–2016	−0.86	38.8–34.4	−11.3
**Fatal assault (homicide)**								
Total, ≥60	N/A	19,059 (100.0)	2.25 (2.22–2.28)	1	2002–2014	−1.14**	2.42–2.11	−12.8
					2014–2016	4.66	2.11–2.31	9.5
Men, ≥60	N/A	11,872 (62.3)	3.16 (3.10–3.22)	1	2002–2010	−2.26**	3.54–2.95	−16.7
					2010–2016	1.17**	2.95–3.16	7.1
Women, ≥60	N/A	7,187 (37.7)	1.53 (1.49–1.57)	0	2002–2016	−0.76**	1.61–1.45	−9.9
Men, 60–69	N/A	7,216 (60.8)	3.58 (3.50–3.66)	1	2002–2013	−1.32**	3.83–3.31	−13.6
					2013–2016	6.16**	3.31–3.95	19.3
Men, 70–79	N/A	3,167 (26.7)	2.76 (2.66–2.86)	0	2002–2016	−2.17**	3.23–2.38	−26.3
Men, ≥80	N/A	1,489 (12.5)	2.48 (2.35–2.61)	0	2002–2016	−2.00**	2.89–2.18	−24.6
Women, 60–69	N/A	3,108 (43.2)	1.40 (1.35–1.45)	0	2002–2016	−0.52	1.46–1.35	−7.5
Women, 70–79	N/A	2,107 (29.3)	1.47 (1.41–1.53)	0	2002–2016	−0.65	1.55–1.42	−8.4
Women, ≥80	N/A	1,972 (27.4)	1.87 (1.79–1.95)	0	2002–2016	−0.86**	1.99–1.76	−11.6

**Abbreviations:** APC = annual percentage change; CI = confidence interval; N/A = not applicable.

* Based on injuries treated in emergency departments. Excludes nonfatal sexual assault cases.

^†^ The nonfatal estimate of injuries was generated from the sample of cases in the National Electronic Injury Surveillance System–All Injury Program. Data are weighted by the inverse probability of sample case selection to produce national estimates. The fatal national estimate of injuries is the actual total homicide cases reported to the National Vital Statistics System in the United States. This total is not estimated from a sample.

^§^ Estimates might not sum to total because of rounding.

^¶^ Rate per 100,000 population.

** Statistically significant regression results (p<0.05).

Nonfatal assault rates among older adults did not change from 2002 to 2008 but increased 53.1% from 2008 to 2016 in the overall sample (Table) (Figure 1). For the entire 15-year period, the nonfatal assault rate increased 75.4% (from 77.7 to 136.3) among men, and from 2007 to 2016, 35.4% (from 43.8 to 59.3) among women. Among adults aged 60–69 years, the nonfatal assault rate against men increased 89.2% (100.5 to 190.1) and against women, increased 37.5% (55.0 to 75.6) from 2002 to 2016.

**FIGURE 1:**
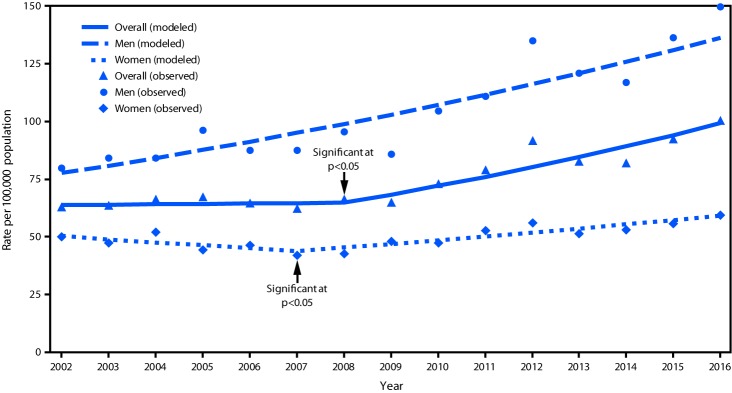
Nonfatal observed and modeled assault injury rates*^,†,§^ among adults aged ≥60 years treated in hospital emergency departments, by sex — United States, 2002–2016 * Excluding sexual assault. ^†^ Joinpoint regression analysis was used to determine annual percentage change (APC) with statistically significant trend and significant joinpoints. ^§^ Overall: APC = 5.47 (2008–2016); men: APC = 4.10 (2002–2016); women: APC = 3.41 (2007–2016).

From 2002 to 2014, the overall estimated homicide rate declined 12.8% (from 2.42 to 2.11) (Table) (Figure 2) and declined 9.9% among women across the entire study period. The rate among men declined 16.7% from 2002 to 2010, but then increased 7.1% (from 2.95 to 3.16) from 2010 to 2016. From 2013 to 2016, an increase of 19.3% (from 3.31 to 3.95) was observed among men aged 60–69 years (Table). Homicide rates declined in all other age groups of men, and among women aged ≥80 years the rate declined 11.6% from 2002 to 2016.

**FIGURE 2:**
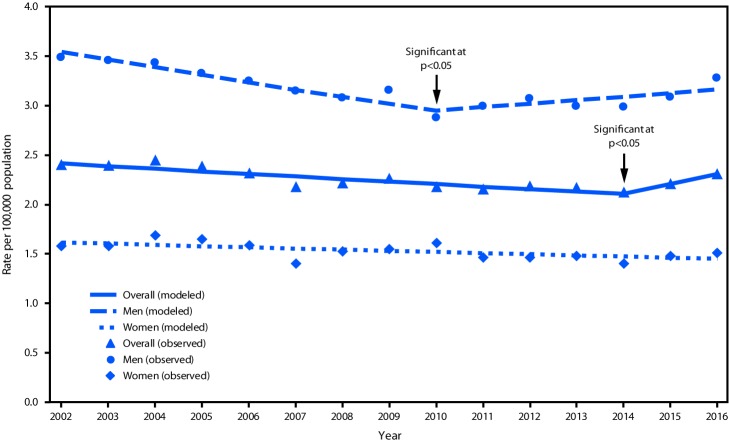
Observed and modeled homicide rates*^,†^ among adults aged ≥60 years, by sex — United States, 2002–2016 * Joinpoint regression analysis was used to determine annual percentage change (APC) with statistically significant trend and significant joinpoints. ^†^ Overall: APC = −1.14 (2002–2014); Men: APC = −2.26 (2002–2010), APC = 1.17 (2010–2016); Women: APC = −0.76 (2002–2016).

Nonfatal assaults and homicides differed with respect to mechanisms used in assaults. Most (86.6%) nonfatal assaults were related to being intentionally struck or hit (e.g., with hand, foot, or object). Firearms were the most common weapons used in homicides (42.2%). Other homicide mechanisms included cutting/piercing (14.8%) and suffocation (6.4%).

## Discussion

During this study period, the older adult population (persons aged ≥60 years) in the United States grew nearly 3% annually, approximating global projections (*2*); in addition, the nonfatal assault rate among this group grew approximately 4% annually on average. The estimated number of nonfatal assaults nearly doubled during this period, and the number could double again by 2030 if both of the growth rates continue. Men aged 60–69 years are at highest risk for nonfatal assaults and homicide victimization, and rates in this group are climbing. The homicide rate remained relatively unchanged or declined for all other older adult demographic groups.

A study examining victim-perpetrator relationships in nonfatal assaults among older adults using NEISS-AIP data found an estimated 58% of perpetrators had a relationship (e.g., familial, acquaintance) with the victim (*7*). Findings on victim-perpetrator relationships among older adults experiencing homicide incidents (excluding homicide-suicide incidents) collected in 32 states provided by the National Violent Death Reporting System for 2016 indicated approximately half (46%) of homicides were perpetrated by a spouse/intimate partner, parent, child, other relative, or friend/acquaintance.** These cases potentially meet CDC’s definition of elder abuse (*4*), suggesting a need for prevention and support services for older adults faced with family- or acquaintance-perpetrated assault.

High rates of nonfatal assaults and homicides among men aged 60–69 suggest this group might be particularly vulnerable to violence. Research is needed to understand why assault and homicide rates are highest among the youngest, and presumably healthiest, group of older adult men. Further exploration of mechanism of assault, perpetrators involved, and incident circumstances by age group might illuminate findings.

Collectively, these findings highlight the need to strengthen violence prevention among older adults. Unfortunately, few strategies have been rigorously evaluated (*8*). Emergency medical services and ED providers are positioned to identify assault cases among older adults and refer victims to support services to prevent subsequent violence (*3*). However, one study found that many ED providers either missed or did not respond to cases of older adult abuse because they lacked knowledge or time required to conduct assessments or were uncertain about how to respond (*9*). Incorporating geriatric specialists in EDs might help link clinical care to service referrals.

The findings in this report are subject to at least four limitations. First, nonfatal injury rates are underestimated because this study only included persons treated in EDs. Second, nonfatal injury data were coded by trained abstractors, and details of injuries can vary across medical records, resulting in coding inaccuracies. However, NEISS-AIP ED visit findings were consistent with those from other large databases such as the Healthcare Cost and Utilization Project.^††^ Third, descriptive characteristics of precipitating and preceding circumstances, victim-perpetrator relationships, and mechanisms used were limited in NEISS-AIP and NVSS. Finally, trends in nonfatal assault cases could not be examined by race/ethnicity because of substantial missing race/ethnicity data in NEISS-AIP. However, homicide rates by race/ethnicity are available online.^§§^ Among older adults, 2002–2016 average crude homicide rates were highest among non-Hispanic blacks, non-Hispanic American Indian/Alaskan Natives, and Hispanics. Racial/ethnic minorities experience a disparate prevalence of violent injury and homicide (*10*). Inequities start early in life and can result from disproportional exposure to residential segregation, concentrated disadvantage, limited educational and employment opportunities, and other conditions that amplify the risk of experiencing violence (*10*). Reductions in systemic inequities could diminish violence against persons of all ages and races, including older adults.

Violence against older adults is an emerging and underreported public health problem. EDs might be promising settings to identify older adults at risk for violence and treat and support those already affected (*3*). Additional information on types, signs, and circumstances of violence among older adults, as well as resources to help victims, is available at https://www.cdc.gov/violenceprevention/pdf/em-factsheet-a.pdf.

**Summary**

**What is already known about this topic?**

The older adult U.S. population is growing faster than are younger populations, yet violence against older adults has received little attention.

**What is added by this report?**

Fifteen-year trends in nonfatal assaults and homicides among adults aged ≥60 years were examined using National Electronic Injury Surveillance System–All Injury Program and National Vital Statistics System data. The estimated nonfatal assault rate increased 75.4% among men (2002–2016) and 35.4% among women (2007–2016). The estimated homicide rate for men increased 7.1% from 2010 to 2016.

**What are the implications for public health practice?**

Violence against older adults is a growing problem, particularly among men. Emergency departments might be positioned to help prevent violence among this group.

## Footnotes

* https://icd.who.int/browse10/2016/en.

† https://www.cdc.gov/nchs/nvss/bridged_race.htm.

§ https://surveillance.cancer.gov/joinpoint/.

¶ Reported rates during joinpoint ranges and percent rate changes are based on models from joinpoint regression analysis.

** https://www.cdc.gov/injury/wisqars/nvdrs.html.

†† https://www.hcup-us.ahrq.gov/.

§§ Homicide data, as well as nonfatal assault data provided by NEISS-AIP, can be accessed using CDC’s Web-based Injury Statistics Query and Reporting System (WISQARS). https://www.cdc.gov/injury/wisqars/dataandstats.html.
